# Profile distribution of CO_2_ in an arid saline-alkali soil with gypsum and wheat straw amendments: a two-year incubation experiment

**DOI:** 10.1038/s41598-018-30312-0

**Published:** 2018-08-09

**Authors:** Junyi Wang, Xiujun Wang, Jiaping Wang

**Affiliations:** 1 College of Global Change and Earth System Science, Beijing Normal University, Beijing, 100875 China; 2 College of Agriculture, Shihezi University, Shihezi, 832000 China

## Abstract

Adding gypsum and/or straw is a common practice for ameliorating saline-alkali soils. However, the effect of amendment on soil CO_2_ is poorly known. An incubation experiment was conducted for over two years in a saline-alkali soil of Yanqi Basin, which included four treatments: control, gypsum addition (Ca), wheat straw addition (S) and gypsum-wheat straw combination (Ca+S). We continuously monitored soil CO_2_ concentration, temperature and moisture at 15, 30, 45 and 60 cm. There was a clear seasonality in soil CO_2_ under all four treatments, which was generally similar to those in soil temperature and moisture. Straw addition led to a significant increase in soil CO_2_ over 0–60 cm in summer. While there was a significant increase of soil CO_2_ with gypsum addition only, soil CO_2_ significantly decreased with the addition of gypsum and straw (relative to straw addition only) during autumn and winter in 2014. Interestingly, integrated soil CO_2_ was lowest in soil profile under the Ca+S treatment during winter and spring. Our study implies that different amendments of organic matter and gypsum may result in various responses and interactions of biological, chemical and physical processes, with implications for the carbon cycle in saline-alkaline soils of arid region.

## Introduction

Saline-alkali soil is widely distributed in arid and semiarid areas due to extremely low precipitation and strong evaporation^1,2^. In the arid area of northwest China, saline-alkali soil covers 36,000 km^2^, accounting for 36% of the national’s total area with salt affected soil^3^. Saline-alkali soil with toxic ions and adverse growth environments greatly inhibits nutrient cycling, especially for agricultural ecosystem, which often results in a decrease of crop yields^1,4,5^. Improvement of saline-alkali soil is one of the effective measures to enhance agricultural production.

Amendments with organic and chemical materials are a common practice to ameliorate saline-alkali soils^6–10^. Extensive studies have showed that application of organic matter, such as crop straw, biochar and green manure, can improve soil fertility and physicochemical properties^11–14^. Similarly, gypsum as a common chemical amendment can improve soil structure and irrigation condition by reducing exchangeable sodium percentage^3,15,16^.

A number of studies have demonstrated that adding organic materials significantly increases organic carbon content and microbial activities, thereby enhancing soil CO_2_ production and emission^17,18^. Some studies indicated gypsum addition has no significant influence on soil CO_2_ emission, due to small change in labile carbon pool or soil microbial biomass^19,20^. However, Kaur *et al*.^21^ found an increase of microbial biomass carbon in gypsum-amended soil as a result of improvement in soil physicochemical environment. There are limited studies of CO_2_ emission in saline-alkali soil with combination of organic matter and gypsum amendments. A 12-weeks incubation experiment by Wong *et al*.^20^ showed that cumulative CO_2_ emission was higher in soil with the addition of both kangaroo grass and gypsum than with addition of kangaroo grass only. On contrary, Schultz *et al*.^22^ reported that CO_2_ emission was lower under the combination of biochar and gypsum relative to biochar addition alone during an ~8-weeks incubation experiment.

Studies have shown that soil CO_2_ emission is regulated by soil CO_2_^23,24^. Usually, there exists a positive correlation between soil CO_2_ emission and CO_2_ concentration, e.g., a linear relationship in a forest soil^25^. However, the relationship may change because environmental conditions (e.g., temperature and precipitation) have influences on CO_2_ diffusion in soil profile. For example, there is evidence of a pause in CO_2_ emission following rain events^26^. On the other hand, soil CO_2_ directly reflects a balance of the sources and sinks associated with the biological, chemical and physical processes. Therefore, studying the variability of soil CO_2_ is critical to understanding the influencing mechanisms of amelioration on the carbon cycle in saline-alkali soils.

Soil CO_2_ dynamics is largely affected by the rate of CO_2_ production. Soil organic matter decomposition, as a main process of CO_2_ production, largely depends on the availability of substrate and microbial activity that is influenced by environment conditions such as temperature, moisture and salinity^27–30^. On the other hand, there are studies linking soil CO_2_ production and consumption with carbonate in arid zone in recent decade, e.g., carbonate dissolution leading to a decrease of soil CO_2_, and carbonate precipitation causing CO_2_ increase^31–33^. Soil CO_2_ dynamics is also influenced by diffusion, i.e., the main process of CO_2_ from soil to the atmosphere, which is related to CO_2_ concentration gradient and soil properties, such as porosity^23,34^.

Saline-alkali soils in the arid and semi-arid land of Northwest China contain a large amount of soil carbonate^35^. Thus, soil CO_2_ may be influenced by both biotic and abiotic processes, following amendments with gypsum and/or wheat straw in saline-alkali soil. However, little is done to evaluate the dynamics of soil CO_2_ in arid saline-alkali soil following the application of gypsum and wheat straw. Here, we hypothesize that (1) application of gypsum could lead to an increase in soil CO_2_ (due to enhanced carbonate precipitation), and (2) application of organic material and gypsum together would induce a larger increase of soil CO_2_ than any single amendment. The objective of this study is to evaluate the impacts of amelioration on soil CO_2_ dynamics, and to explore the underlying mechanisms regulating the variability of soil CO_2_ in an arid saline-alkali soil. We conducted an incubation experiment for more than two years in Yanqi Basin, Xinjiang, which consists of various amendments.

## Results

### Variations of soil environmental factors

**Figure 1:**
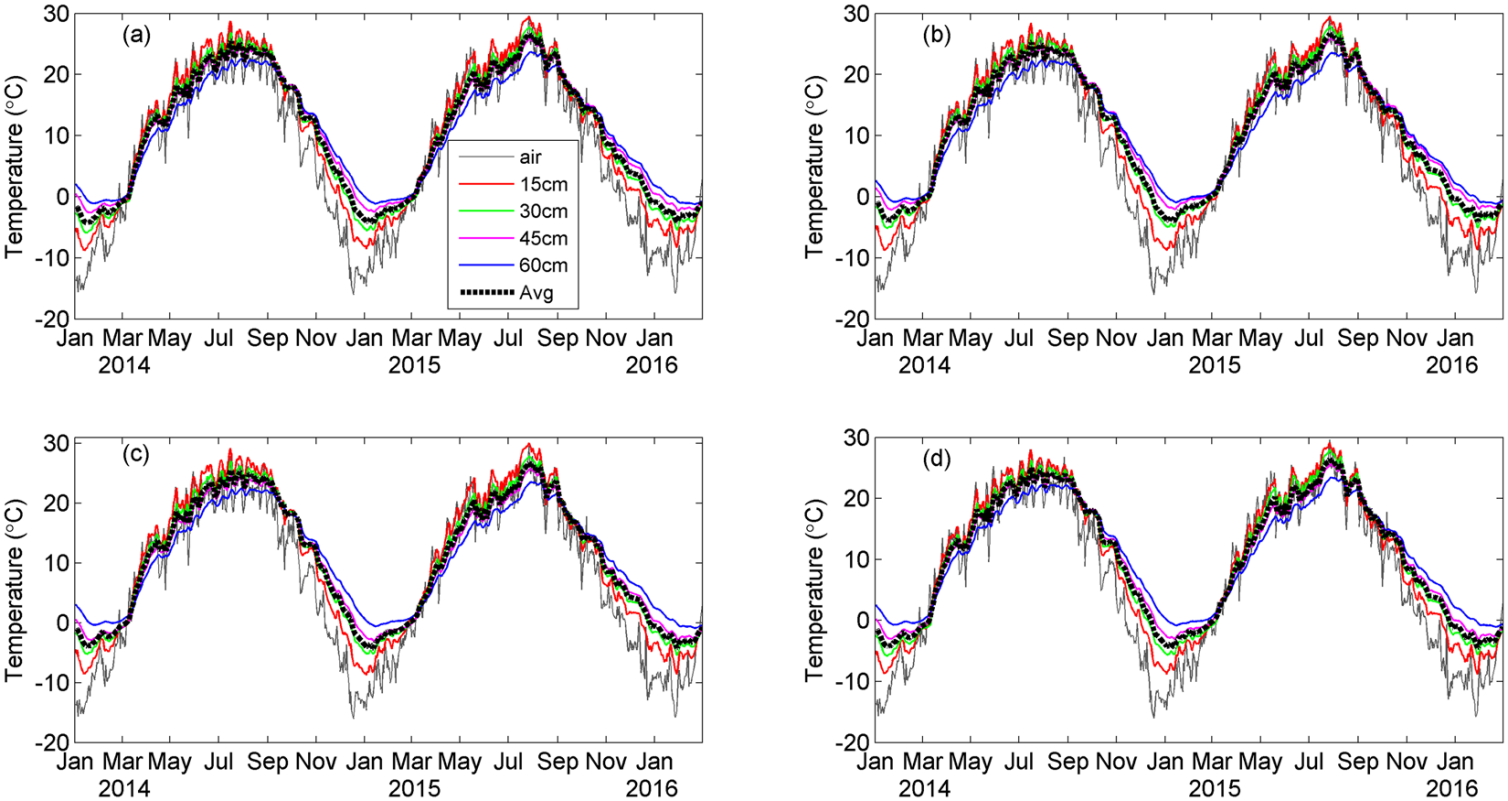
Daily temperature in air and at various depths in soil under (**a**) control, (**b**) Ca, (**c**) S, and (**d**) Ca+S treatments. The black dotted line represents the average value over 0–60 cm. Air temperature data were obtained from a local mini weather station ~10 km away. The figure was generated using Matlab R2012b software (https://www.mathworks.com/matlab).

Temperature showed obvious seasonal variation with similar pattern in air and soil under four treatments in 2014 and 2015 (Fig. 1), displaying a curve with single peak (the maximum) in July. In soil, the annual variation of temperature is the largest at 15 cm and the smallest at 60 cm. Average soil temperature above 60 cm showed no significant difference under all treatments in spring and summer between 2014 and 2015 (P > 0.05). However, soil temperature in autumn (winter) was significantly higher (lower) in 2014 than in 2015 (P < 0.001, Table 1). There was no significant difference in average soil temperature among four treatments in both years (P > 0.05).

**Table 1:** Mean air temperature and soil temperature (averaged over 0–60 cm) for spring, summer, autumn, winter and whole year in 2014 and 2015.

Variables	Treatments	Year	Spring	Summer	Autumn	Winter	Whole year
Air temperature		2014	11.5	21.8	8.2	−8.3	8.4
(ºC)		2015	12.5	22.3	8.2	−7.9	8.8
Soil temperature	control	2014	11.0	23.0	13.9	−1.7	11.6
(ºC)		2015	11.3	22.9	12.7	−1.0	11.5
	Ca	2014	11.0	23.0	14.1	−1.3	11.7
		2015	11.3	22.9	12.7	−0.6	11.6
	S	2014	11.3	23.2	14.2	−1.5	11.9
		2015	11.4	22.9	12.6	−0.8	11.6
	Ca+S	2014	10.9	22.7	13.8	−1.8	11.5
		2015	11.1	22.6	12.5	−1.1	11.3

**Figure 2:**
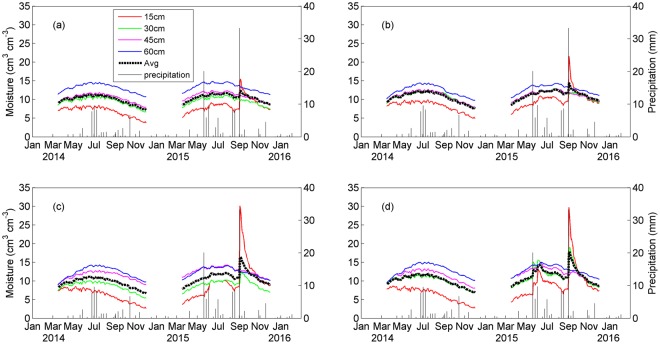
Soil volumetric moisture content at various depths (no values when temperature below 0 °C) under (**a**) control, (**b**) Ca, (**c**) S, and (**d**) Ca+S treatments. The vertical lines denote weekly precipitation data that were obtained from a local mini weather station ~10 km away. The figure was generated using Matlab R2012b software (https://www.mathworks.com/matlab).

The variations of precipitation and soil moisture during 2014–2015 are shown in Fig. 2. Precipitation in 2014 mainly occurred in summer. Averaged moisture above 60 cm revealed a small increase or little change in spring, with the maximum in summer and followed by a decreasing trend in autumn under all four treatments. The occurrence of rainfall events dramatically increased in 2015, and total precipitation (113.0 mm) in 2015 was greater than in 2014 (53.1 mm, Table 2). Correspondingly, soil moisture showed a significant increase in summer and autumn (P < 0.001), but not in spring under all treatments. Especially, following the large amount of rainfall (34.1 mm) from August 30 to September 2, soil water content increased sharply and reached the maximum, i.e., 15.5%, 21.6%, 30.2% and 29.7% at 15 cm under the control, Ca, S and Ca+S treatments, respectively. Interestingly, soil moisture was significantly higher under the Ca (Ca+S) treatment than control (S) treatment in both years (P < 0.001).

**Table 2:** Precipitation and mean soil moisture (0–60 cm) for spring, summer, autumn, winter and whole year in 2014 and 2015.

Variables	Treatments	Year	Spring	Summer	Autumn	Winter	Whole year
Precipitation		2014	4.5	32.8	14.1	1.7	53.1
(mm)		2015	28.3	54.7	27.1	2.9	113.0
Moisture	control	2014	10.2	10.9	8.7	nd^a^	9.9
(%)		2015	10.2	11.3	10.3	nd	10.6
	Ca	2014	10.8	11.9	9.2	nd	10.6
		2015	10.4	12.1	11.3	nd	11.3
	S	2014	9.7	10.7	8.3	nd	9.6
		2015	9.6	11.6	11.5	nd	11.0
	Ca+S	2014	10.5	11.4	8.6	nd	10.1
		2015	10.3	12.2	11.6	nd	11.4

^a^nd: no data.

### Temporal variation of soil CO_2_

**Figure 3:**
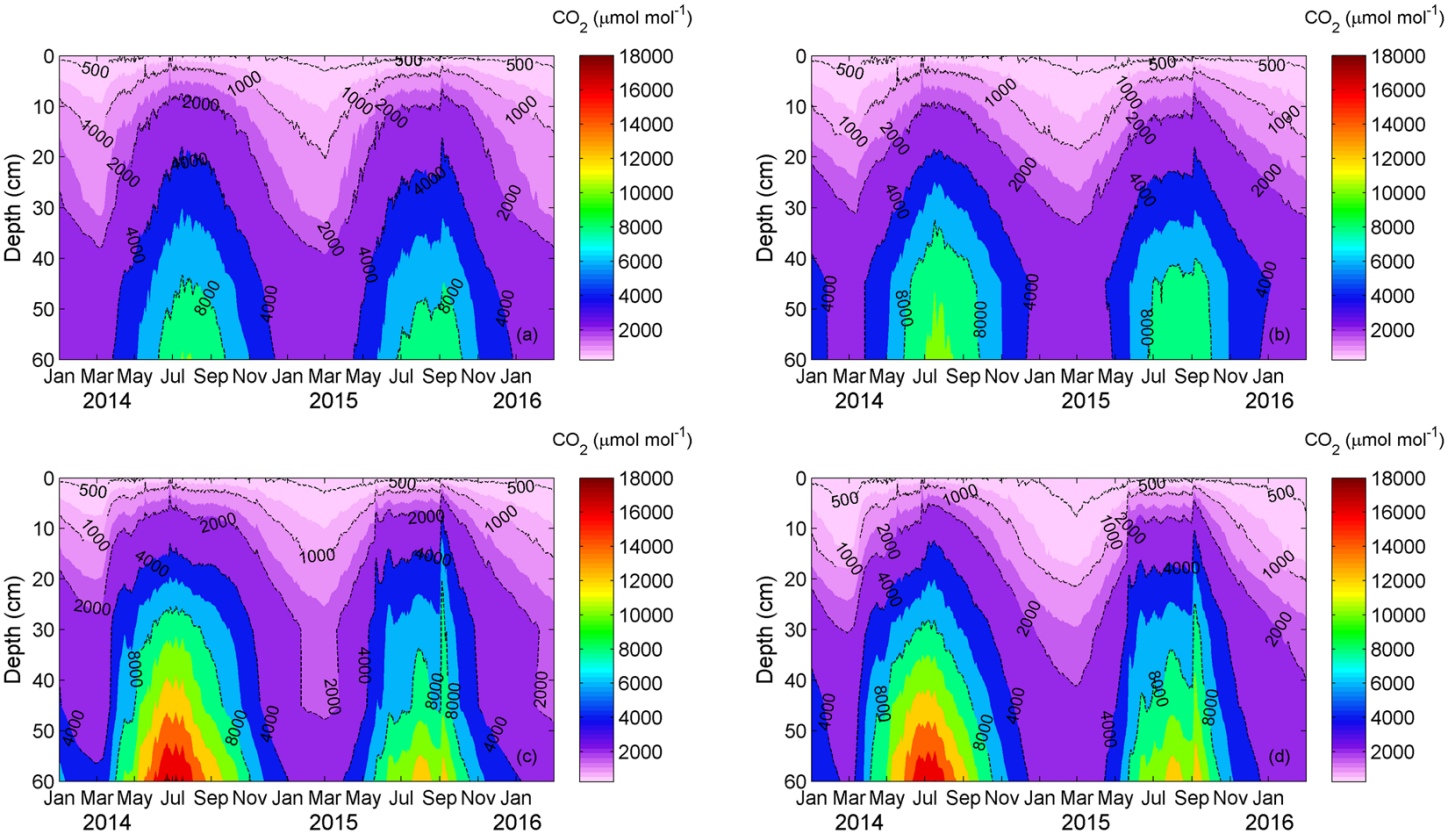
Vertical-temporal variation of soil CO_2_ under (**a**) control, (**b**) Ca, (**c**) S, and (**d**) Ca+S treatments. The figure was generated using Matlab R2012b software (https://www.mathworks.com/matlab).

Soil CO_2_ concentration revealed clear spatial and temporal variations over the period of 2014–2015 (Fig. 3), showing a gradual increase with depth under all four treatments. Soil CO_2_ generally displayed an increasing trend in spring and summer, followed by a decreasing trend in autumn and winter. There were considerable differences in the seasonal pattern between 2014 and 2015. In 2014, soil CO_2_ reached peak in July under the control and Ca treatments, which was similar to that in soil temperature. However, the maximum of soil CO_2_ appeared a month earlier than that of temperature under the S and Ca+S treatments. In 2015, soil CO_2_ showed some fluctuations during the period of June-October under all treatments, which was associated with some rainfall events. For example, following the heavy rainfall from August 30 to September 2, average CO_2_ concentration over 0–60 cm increased by 737, 715, 2370 and 2244 μmol mol^−1^ under the control, Ca, S and Ca+S treatments, respectively. The most noticeable decrease under the Ca, S and Ca+S treatments happened in July and August of 2015, comparing with 2014. During this period, averaged CO_2_ concentrations over 0–60 cm reduced by 14%, 20% and 18% under the Ca, S and Ca+S treatments, respectively. Apparently, soil CO_2_ was significantly higher under the S and Ca+S treatments than control and Ca treatments, especially in summer. For example, average soil CO_2_ with straw addition increased by ~38% and ~17% in 2014 and 2015, respectively. On the other hand, soil CO_2_ under the Ca treatment was slightly higher than under the control treatment in both years.

### Differences of soil CO_2_ among treatments

**Figure 4:**
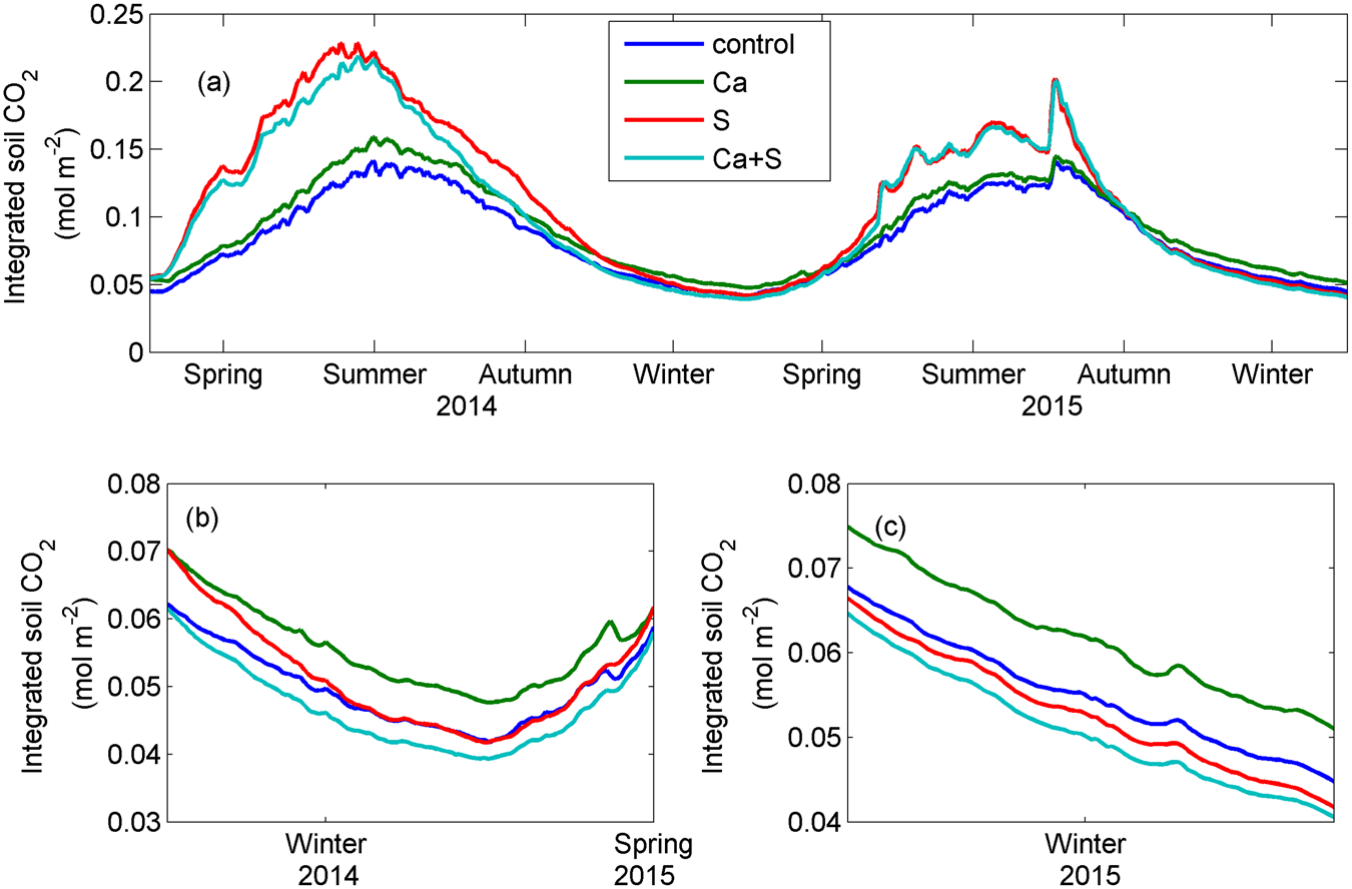
Time series of integrated soil CO_2_ over 0–60 cm for the period of (**a**) 2014–2015, (**b**) winter-early spring in 2014/2015, and (**c**) winter in 2015. The figure was generated using Matlab R2012b software (https://www.mathworks.com/matlab).

**Figure 5:**
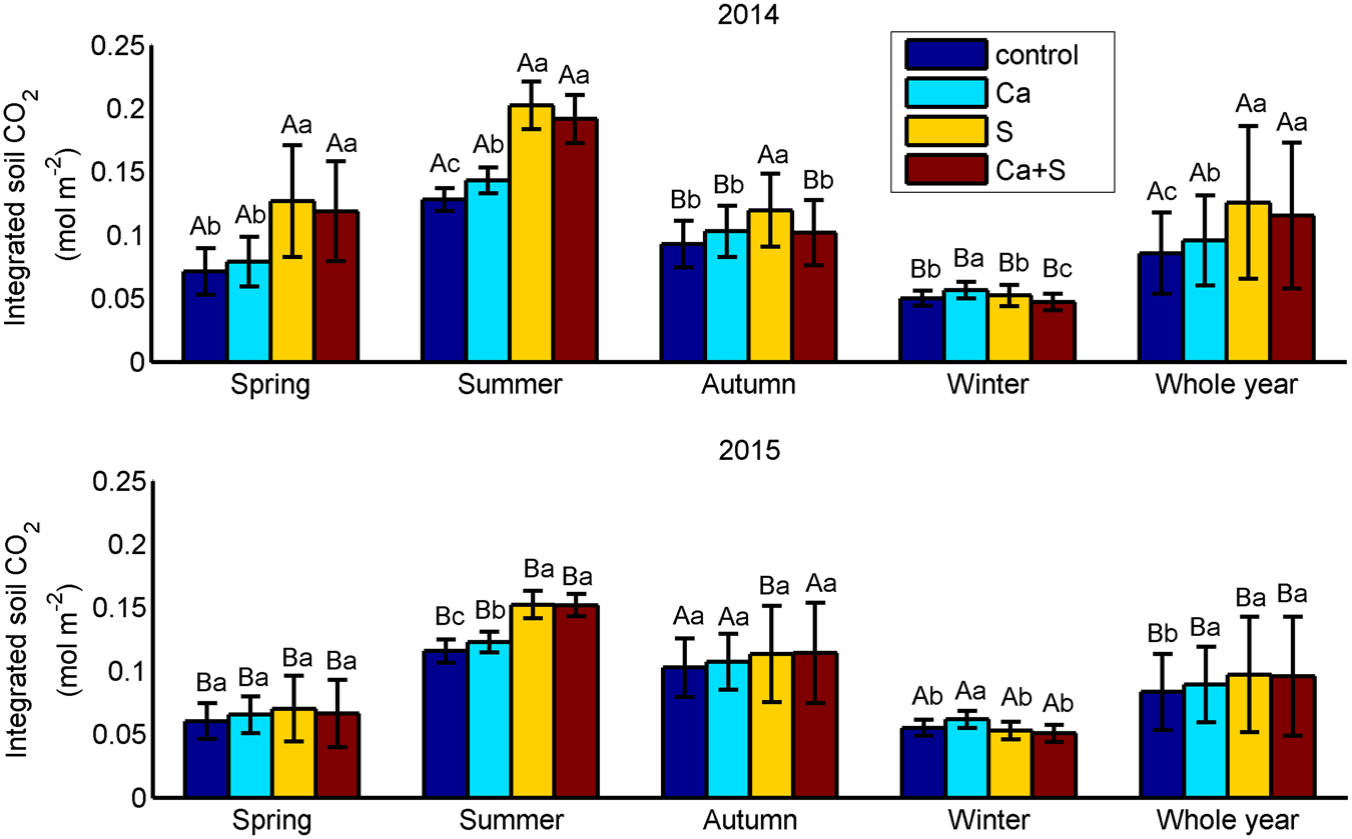
Means of integrated soil CO_2_ over 0–60 cm for spring, summer, autumn, winter and whole year in 2014 (upper panel) and 2015 (lower panel) under the control, Ca, S and Ca+S treatments. Values with different letters above error bars (i.e., standard deviations) are significantly different at 0.05 level. Uppercase letters denote differences between 2014 and 2015 under the same treatment, and lowercase letters among different treatments for each season or whole year. The figure was generated using Matlab R2012b software (https://www.mathworks.com/matlab).

To further evaluate the effects of amendments on soil CO_2_, we calculated total soil CO_2_ accumulated over the 0–60 cm (Fig. 4). Clearly, integrated soil CO_2_ was significantly higher during spring-summer in 2014 than in 2015 (P < 0.001), but not during autumn-winter (Fig. 5). There was a significant increase in soil CO_2_ over 0–60 cm with wheat straw addition in summer for both years (P < 0.001). Interestingly, integrated soil CO_2_ under the Ca treatment was significantly higher than that under the control treatment (P < 0.001) whereas integrated soil CO_2_ was obviously lower under the Ca+S treatment than S treatment in 2014. There was no significant difference between the S and Ca+S treatments during spring and summer, but a significant difference during autumn and winter (P < 0.001). Surprisingly, integrated soil CO_2_ was highest under the Ca treatment but lowest under the Ca+S treatment in winter of both years (Fig. 4b,c). For example, in 2014, integrated soil CO_2_ over 0–60 cm was 0.057 mol m^−2^ under the Ca treatment, which was significantly higher than those under the control (0.050 mol m^−2^), S (0.052 mol m^−2^) and Ca+S (0.047 mol m^−2^) treatments. Overall, there were relatively small differences in integrated soil CO_2_ over 0–60 cm among four treatments during spring and autumn, especially in 2015.

## Discussion

### Regulations of environmental conditions

**Figure 6:**
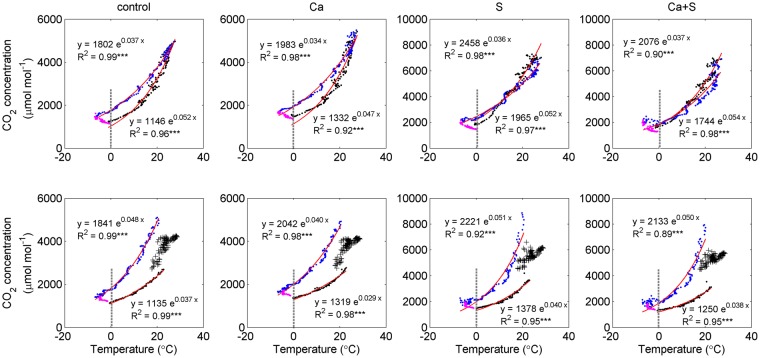
The relationship between mean soil CO_2_ concentration and temperature over 0–30 cm under the control, Ca, S and Ca+S treatments in 2014 (upper panel) and 2015 (lower panel). The black (blue) dots were for the period of warming (cooling). The pink dots were for the warming period at the end of winter. Red lines are fitted curves. The vertical dashed lines indicate the positions of 0 °C. ***significant at P < 0.001. The figure was generated using Matlab R2012b software (https://www.mathworks.com/matlab).

Temperature and moisture are the most important soil environmental factors^36,37^. Our analyses showed that soil CO_2_ concentration was exponentially related to soil temperature under all four treatments (P < 0.001) with exception at the end of the winter and the period affected by rain (Fig. 6). The relationship was mainly attributed to the influence of temperature on organic matter decomposition^38–40^. However, there were significant differences in the soil CO_2_-temperature relationship between warming period and cooling period, and between 2014 and 2015, indicating that other processes (e.g., diffusion) had influences on soil CO_2_. There was a significant difference in the seasonality of precipitation between 2014 and 2015, which could explain the large differences in the soil CO_2_-temperature relationship between these two years. For example, the intense rainfall events in the summer of 2015 led to relatively high levels of soil CO_2_ in soil profiles, which was similar to the finding by Tang *et al*.^41^.

Our study showed that the soil CO_2_-temperature relationship was inconsistent under freezing condition (temperature below 0 °C). There was a robust negative relationship (P < 0.001) between soil CO_2_ and temperature during warming (pink dots in Fig. 6). However, the relationship during cooling under freezing condition was generally positive under the treatments without straw addition but negative with straw amendments (Fig. 6). Pumpanen *et al*.^42^ reported that temperature drop below 0 °C resulted in soil CO_2_ elevation during late-winter and early-spring in a Scots pine forest soil, which was consistent with our findings under the straw amendments during cooling. Given little CO_2_ production under low temperature, the discrepancies in the response of soil CO_2_ to temperature change under freezing condition may result from the differences in soil properties, e.g., porosity and texture that influence CO_2_ diffusivity, and their responses to temperature change^43,44^.

**Figure 7:**
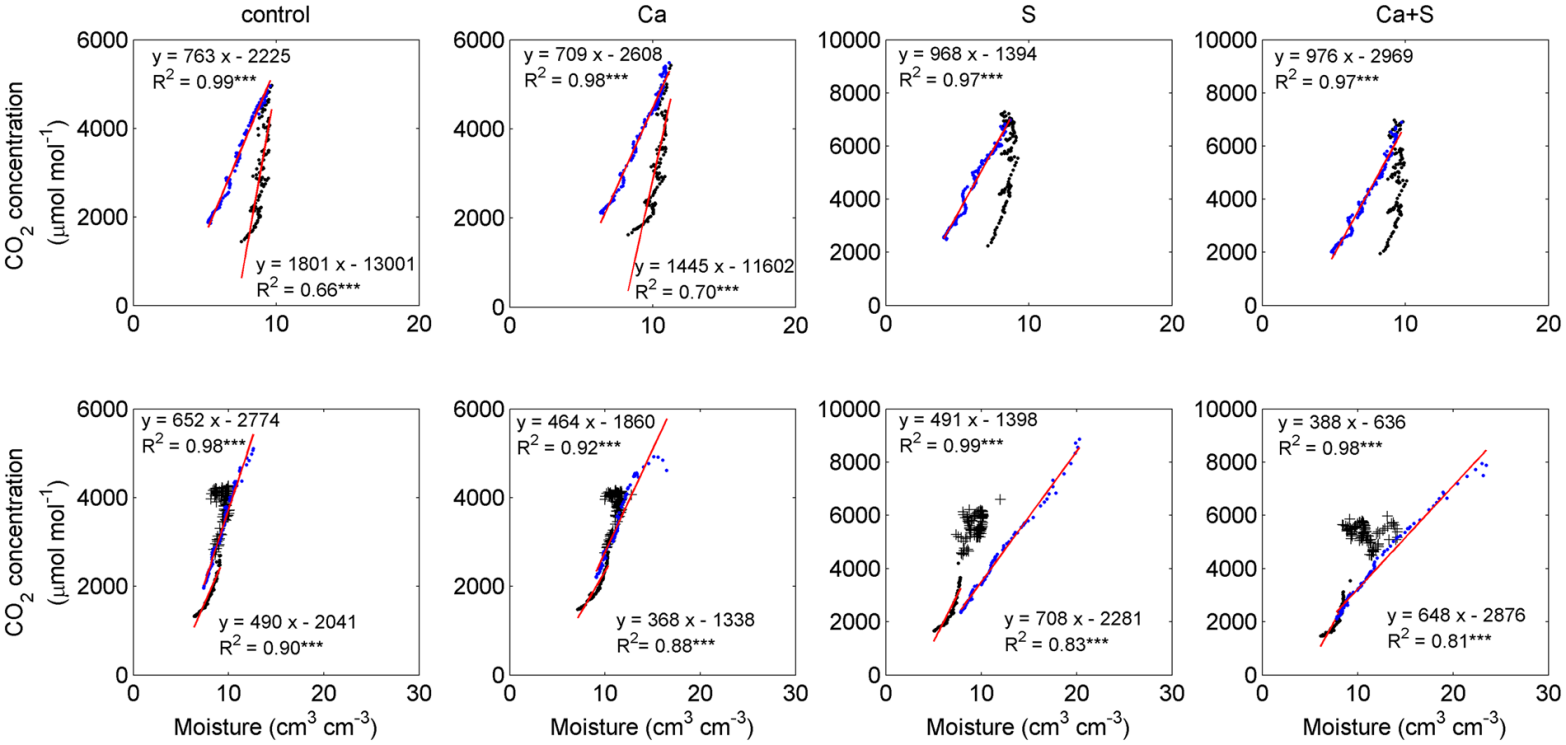
The relationship between mean soil CO_2_ concentration and moisture over 0–30 cm under the control, Ca, S and Ca+S treatments in 2014 (upper panel) and 2015 (lower panel). The black (blue) dots were for the period of warming (cooling). Red lines are fitted curves. ***significant at P < 0.001. The figure was generated using Matlab R2012b software (https://www.mathworks.com/matlab).

Our study showed a linear positive relationship between soil CO_2_ and moisture (P < 0.001) with exception during the period affected by rain events (Fig. 7). During warming period in 2014, there was no significant relationship between soil CO_2_ and moisture under the S and Ca+S treatments. During this period, temperature may be the main factor influencing soil CO_2_.

There was evidence that soil CO_2_ increased with moisture, especially following significant rainfall^45,46^, which might result in enhancement in organic matter decomposition and reduction in CO_2_ diffusion due to a decrease in air-filled porosity^25,47^. However, the effect of precipitation on soil CO_2_ concentration may vary, which depends on the timing and intensity of precipitation, and soil moisture^41,48^. Our study showed that during the intense rainfall events, there was irregular change in soil CO_2_ that had no significant relationship with either temperature or moisture. Apparently, the influence of rainfall on the dynamics of soil CO_2_ is complicated, owing to various responses in associated biological, chemical and physical processes.

### Influences of individual amelioration

Soil CO_2_ was significantly affected by the addition of gypsum and/or wheat straw. As expected, application of wheat straw significantly increased soil CO_2_, owing to the increase in substrate availability and microbial activity, thus enhanced CO_2_ production^49,50^. Many studies have reported similar findings, i.e., an increase of CO_2_ emission as a result of crop straw amendment in farmland or forest soils^51–53^.

Our study showed an increase of CO_2_ concentration in gypsum-amended soil (relative to the control treatment), which may be associated with the increase in moisture (Table 2). The effects of increased moisture on soil CO_2_ would include biological (enhancing microbial decomposition of organic matter), and physical (reducing porosity thus less diffusion) processes^25,47^. On the other hand, soil CO_2_ might be also influenced by chemical processes in arid zone, i.e., dissolution/precipitation of carbonate controlled by carbonate–bicarbonate equilibria^54^:

1 $$C{a}^{2+}+2HC{O}_{3}^{-}\leftrightarrow CaC{O}_{3}+C{O}_{2}+{H}_{2}O$$

It is clear that a change in related ions concentration, moisture and/or temperature will alter the carbonate dissolution/precipitation equilibrium, which would lead to a change in soil CO_2_ concentration. Apparently, adding Ca^2+^ in soil profile would be beneficial for carbonate precipitation and also CO_2_ creation^15,31^, which could partly explain why soil CO_2_ was the highest under Ca treatment in winter.

While adding Ca^2+^ can lead to changes in chemical reactions/equilibrium^15^, gypsum amendment can also cause changes in soil biological and physical properties^20,22^, which may impact the CO_2_ production and diffusion process thus alter soil CO_2_ dynamics. According to Wong *et al*.^20^, soil amendment with gypsum caused a small decrease in CO_2_ emission, which might be associated with changes in soil properties; there was a large increase of electrical conductivity (EC) due to gypsum addition in their experiment, which would reduce microbial activity and soil CO_2_ production, thus decreased CO_2_ emission from soil to the atmosphere^49^. Similarly, Schultz *et al*.^22^ reported a decrease of CO_2_ emission in gypsum-amended soil, which might result from a decrease in porosity due to an increase in soil bulk density.

### Influences of combined amendments

Given that soil CO_2_ increases under gypsum amendment or straw addition, one would expect that the combination of gypsum and wheat straw addition would induce a further increase in soil CO_2_. However, we found that integrated soil CO_2_ over 0–60 cm was lower under the Ca+S treatment than the S treatment, indicating that there may be changes in environmental conditions and soil properties, which have complicated influences on soil CO_2_ dynamics. Below, we explore the possible underlying mechanisms associated with the combined amendments of gypsum and straw.

Firstly, straw addition can produce acid microenvironment following decomposition, which is beneficial for carbonate dissolution^21,55,56^, and such effect may counteract carbonate precipitation and CO_2_ increase induced by gypsum addition. Secondly, combination of gypsum-straw amendments results in further improvement of soil properties including soil pH and hydraulic conductivity^13^, which would be beneficial for CO_2_ diffusion in soil profile thus emission from soil to atmosphere so less CO_2_ would remain in soil profile. Such situation may be applicable to the Ca+S treatment during winter when soil CO_2_ is the lowest. On the other hand, the excess Ca^2+^ from gypsum can enhance the absorption of organic matter to soil particles, which reduces microbial decomposition thus CO_2_ production. The recent study by Kim *et al*.^57^ indicated that organic matter absorbed to soil aggregates was 14% higher in soil addition with gypsum and rice straw together than rice straw alone.

There have been limited studies that show different results about CO_2_ emission in saline-alkaline soils treated with gypsum and organic materials together^20,22^. Wong *et al*.^20^ reported higher rate of CO_2_ emission due to the application of gypsum and kangaroo grass together, comparing with the addition of kangaroo grass alone. However, Schultz *et al*.^22^ found that there was a decrease in CO_2_ emission under the combined application of biochar and gypsum (relative to biochar addition alone). The different findings in their studies may reflect the large difference in the structure of organic material (grass vs. biochar). In addition, the combination/interaction of organic matter and gypsum may have various influences on soil physicochemical properties with implications for CO_2_ production and emission in saline-alkaline soils.

## Conclusion

The amelioration of saline-alkali soil with gypsum and/or wheat straw had a significant effect on soil CO_2_ in the arid region. Significant increase in soil CO_2_ with wheat straw addition was mainly due to an increase in organic matters’ availability. Gypsum addition led to a small increase in soil CO_2_ concentration, which might result from various biotic and abiotic processes. However, combined application of wheat straw and gypsum resulted in a decrease of soil CO_2_ relative to wheat straw application alone. In particular, integrated soil CO_2_ was lowest in soil profile under the gypsum-straw treatment during winter and spring. While our data showed a generally significant positive relationship between CO_2_ concentration and temperature, the soil CO_2_-temperature relationship was complex under freezing condition. We found that soil CO_2_ increased with moisture, especially following significant rainfall. Further process studies with integrative approaches are needed to better understand the influences of soil ameliorations on physical, chemical and biological processes in saline-alkaline soils, and to investigate the complex impacts of environmental conditions on both soil CO_2_ dynamics and CO_2_ emission.

## Materials and Methods

### Study site description

The field incubation experiment was conducted in Yanqi basin located in the northeast of Tarim basin, Xinjiang, China (86°46′~85°08′E, 41°53′~42°51′N). The climate is continental desert climate, hot and almost 60 percent rain in the summer and relatively cold and dry in winter, and large temperature difference between day and night. The annual average precipitation is less than 80 mm, and the annual average evaporation 2000–2449 mm. The main soil types of this area are brown desert soil and grey brown desert soil, and salinization/alkalization is widespread. Soil pH and EC were 8.1 and 0.8 ms cm^−1^, respectively. Soil organic carbon (SOC) and inorganic carbon (SIC) were 10.9 g(C) kg^−1^ and 22.9 g(C) kg^−1^, respectively, with 8.5% clay, 72.7% silt and 18.8% sand.

### Experiment design

Topsoil (0–30 cm) was collected from the farmland in October 2013, which was passed through a 5-mm sieve (to remove roots and rocks) and mixed thoroughly to ensure the homogeneity. Empty PVC tubes (80 cm high, 50 cm in diameter) were buried in the field (~70 cm deep), then filled with well-mixed soil with or without amendments, namely control (no amendment), Ca (gypsum addition at rate of 1000 Kg/ha), S (wheat straw addition 1.25% (w/w)) and Ca+S (gypsum plus wheat straw addition at the same rates as Ca and S treatments). Wheat straw was cut into 1–2 cm in length. Soil amendments were added at rates in compliance with local agricultural management.

In each tube, we vertically installed CO_2_ sensors (GMT220 series, Vaisala Inc., Finland) to measure soil CO_2_ concentration at 15, 30, 45, 60 cm. All CO_2_ sensors were put inside custom-built steel pipes (same length and 0.5 cm bigger in diameter compared to sensor), the lower ends covered with waterproof-breathable membrane (PUW 867). In addition, one CO_2_ sensor was vertically placed just above the soil surface to monitor atmospheric CO_2_ concentration. Soil temperature and soil volumetric moisture content were monitored by using temperature probes (109, Campbell Scientific Inc., USA) and water content reflectometers (CS616, Campbell Scientific Inc., USA). All data were recorded in the CR1000 data logger (Campbell Scientific Inc., USA).

### Data analysis

In this study, we analyzed data for the period from 1 January 2014 to 29 February 2016, so we could get the mean values for two winters. The Kruskal-Wallis test was used to compare the differences in soil temperature, moisture and CO_2_ concentration among different treatments; the Wilcoxon test was used to compare the difference between two years for individual treatments. The statistical tests were conducted using the SPSS Statistics 20.0 (SPSS Inc., Chicago, IL, USA).

### Data availability

The datasets generated during and/or analyzed during the current study are available from the corresponding author on reasonable request.
